# Tactical Release of a Sexually-Selected Pheromone in a Swordtail Fish

**DOI:** 10.1371/journal.pone.0016994

**Published:** 2011-02-09

**Authors:** Gil G. Rosenthal, Jessica N. Fitzsimmons, Kristina U. Woods, Gabriele Gerlach, Heidi S. Fisher

**Affiliations:** 1 Department of Biology, Texas A&M University, College Station, Texas, United States of America; 2 Centro de Investigaciones Científicas de las Huastecas “Aguazarca”, Calnali, Hidalgo, Mexico; 3 Department of Biology, Boston University, Boston, Massachusetts, United States of America; 4 Marine Biological Laboratory, Woods Hole, Massachusetts, United States of America; University of Maryland, United States of America

## Abstract

**Background:**

Chemical communication plays a critical role in sexual selection and speciation in fishes; however, it is generally assumed that most fish pheromones are passively released since most fishes lack specialized scent glands or scent-marking behavior. Swordtails (genus *Xiphophorus*) are widely used in studies of female mate choice, and female response to male chemical cues is important to sexual selection, reproductive isolation, and hybridization. However, it is unclear whether females are attending to passively produced cues, or to pheromones produced in the context of communication.

**Methodology/Principal Findings:**

We used fluorescein dye injections to visualize pulsed urine release in male sheepshead swordtails, *Xiphophorus birchmanni*. Simultaneous-choice assays of mating preference showed that females attend to species- and sex-specific chemical cues emitted in male urine. Males urinated more frequently in the presence and proximity of an audience (conspecific females). In the wild, males preferentially courted upstream of females, facilitating transmission of pheromone cues.

**Conclusions/Significance:**

Males in a teleost fish have evolved sophisticated temporal and spatial control of pheromone release, comparable to that found in terrestrial animals. Pheromones are released specifically in a communicative context, and the timing and positioning of release favors efficient signal transmission.

## Introduction

A fundamental concern of animal communication is when and where to emit signals. While some signals, like many morphological ornaments, are always “on,” most signals like song, advertisement calls, and motor displays are emitted in a way that optimizes transmission to intended receivers [1]. If signals are costly to produce, or if they incur the risk of eavesdropping by rivals or predators [2] selection should favor tactical signaling. Specifically, signaling should occur preferentially in the presence of intended receivers, i. e. the ‘audience effect,’ [3] and in such a way as to optimize transmission in the physical environment [4].

Such tactical signaling has contributed to the effectiveness of chemical communication across taxa. The highly complex social organization of ants, for instance, is mediated through sophisticated patterns of pheromone release in time and space [5]. In salamanders, release of male sexual pheromones in concert with stereotyped courtship motor patterns stimulates female receptivity [6]. Among aquatic animals, male crustaceans release urine towards opponents during agonistic interactions with other males [7], [8].

Chemical communication is also widespread in fish [9]. While chemosignals are sometimes associated with specialized scent glands [10], [11], release of chemical signals in fish is often hard to identify. However, urine-borne chemicals in fishes can serve a signal function even in the absence of derived morphological structures or visually-apparent behaviors. In goldfish (*Carassius auratus*), steroid conjugates released in the urine are a signal of female sexual receptivity, and females increase urination frequency in the presence of males[12]. In Mozambique tilapia (*Oreochromis mossambicus*), urine-borne signals indicate male social dominance [13], and males increase urination frequency in the presence of sexually receptive females [14].

Swordtails (Poeciliidae: genus *Xiphophorus*) are an important model system in animal communication. Numerous studies have addressed the role of male chemical cues in species recognition [15], [16], [17], [18], [19], [20]. Cues are evolutionarily labile, and phylogenetically more derived signals tend to be more attractive to females [18], [21]. With one exception [21], however, females prefer the scent of conspecific males over heterospecific male scent. In multimodal tests of female mating preferences, olfactory cues are more salient than sexually-dimorphic visual ornaments, and preference for conspecific scent overrides preferences for heterospecific males, even though the latter are more visually conspicuous [15], [22]. Accordingly, chemical cues play a determining role in reproductive isolation between sympatric swordtails. The breakdown of species recognition via chemical cues has led to recent hybridization in natural populations of swordtails[23], [24]. Females also attend to individual variation in male olfactory cues, depending on both male [25] and female [26] nutritional condition.

None of these studies have addressed how and when olfactory cues are released. Given the importance of olfactory information to male mating success, selection should favor signaling strategies that maximize attractiveness to females [4]. In this study, we used laboratory experiments and field observations to study the context of pheromone release in *Xiphophorus.* We collected the urine of male swordtails immediately following courtship events. We showed (1) that urine-based cues are sufficient to elicit female sexual response; (2) that male urine release is dependent on both the presence of and proximity to females; and (3) that males in the wild employ courtship tactics that optimize transmission of urine-borne cues.

## Results

**Figure 1:**
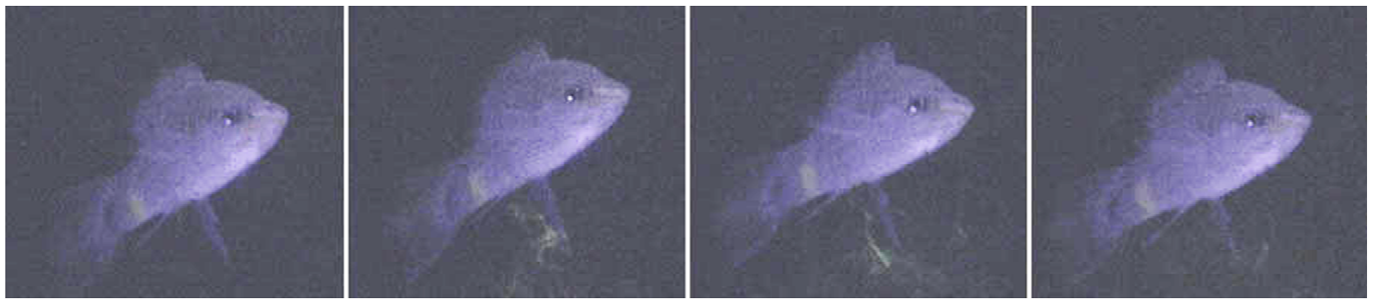
Fluorescein-injected male *X. birchmanni* urinating under ultraviolet light. Still images taken over at 2-second intervals show pulsatile nature of urine release.

**Figure 2:**
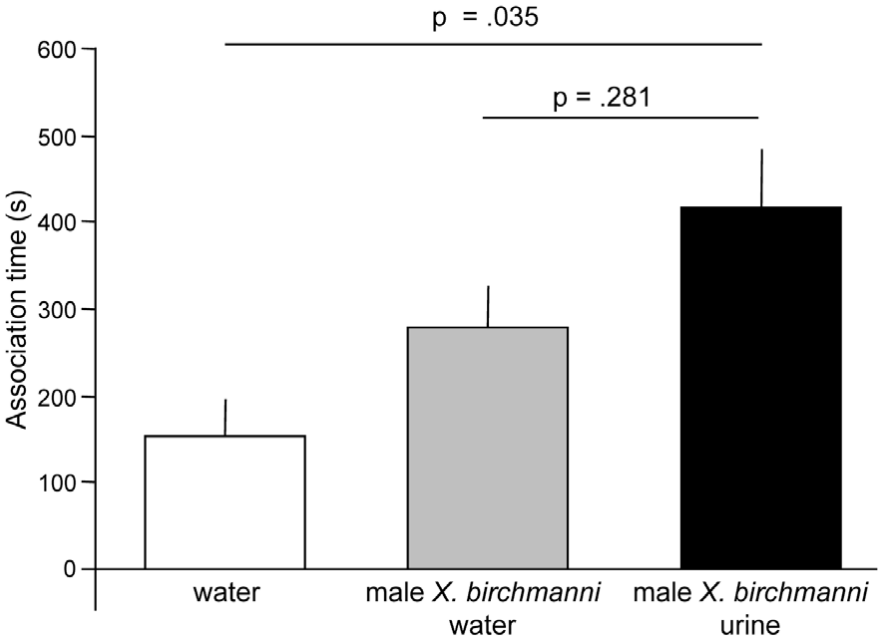
Urine-borne cues elicit female response. (a) Association time (mean + SE) of female *X. birchmanni* in three-way choice tests: conspecific “male water” [16], [17], [18], [22], conspecific male urine, and plain water. *p-*values from Wilcoxon signed-ranks tests are shown.

Males released urine in discrete pulses (Figure 1). Female *X. birchmanni* preferred diluted male *X. birchmanni* urine over a blank control, and failed to discriminate urine from “male water” [16], [17], [18] previously occupied by courting conspecific males (*n* = 15, Friedman test statistic  = 9.39, *p* = 0.009; urine vs. blank control, Wilcoxon signed-rank (WSR) test: *z* = 2.10, *p* = 0.035; urine vs. male water, WSR test: *z* = 1.079, *p* = 0.281; Figure 2). They preferred heterospecific *X. malinche* male urine over conspecific urine (WSR test: *z* = 2.166, *n* = 14, *p* = 0.030), and urine of male conspecifics over that of female conspecifics (WSR test: *z* = 2.803, *n* = 10, *p* = 0.0025).

**Figure 3:**
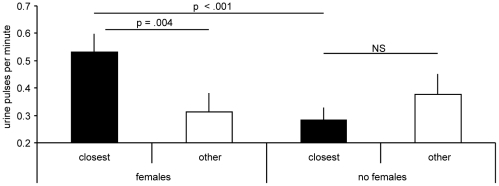
Males prefer to urinate near females. Mean (+ SE) number of male urine pulses per hour within 12.5 cm of the adjacent stimulus aquarium (closest: quadrant 1) and in the remaining 37.5 cm of the male's aquarium (other: quadrants 2–4) with females present and absence. *p-*values from Wilcoxon signed-ranks tests are shown.

Males showed a substantial increase in urination rate when in the presence of females as opposed to when females were absent (WSR test: *z* = 2.073, *n* = 9, *p* = 0.038; Figure 3). This was driven by a marked increase in urination specifically when males were close to females. As expected, males spent much more time in quadrant 1, the area closest to females, when females were present in the adjacent stimulus tank (72% versus 39% when females were absent; WSR test: z = 2.666, *n* = 9, *p* = 0.008). When males were in quadrant 1, urination rate per unit time was significantly higher when females were present (WSR test: *z* = 2.547, *n* = 9, *p* = 0.011). Conversely, urination rates in the remaining quadrants were unaffected by the presence of females, and in fact the numerical trend was in the direction of more urinations when females were absent (WSR test: *z* = 1.244, *n* = 9, *p* = 0.214).

In the wild, 91±4% (mean ± SD) of courtship time in 41 observed interactions was spent upstream of females, within 60 degrees of the prevailing current as estimated by particle flow, significantly more than the 1/6 of courtship time expected by chance if males are courting indiscriminately within a 360° radius of the female (one-sample *t* = 19.35, *P*<.001).

## Discussion

Urine-borne chemical cues afford males the opportunity to control the way signals are emitted in time and space. Male *X. birchmanni*, like female goldfish [12]and male tilapia [13], urinate in discrete pulses lasting up to 5 s (Figure 1). Male urine is sufficient to elicit the same response as the “male water” previously used in olfactory mate-choice experiments in *Xiphophorus* [16], [17], [18], [22]. Female *X. birchmanni* did not distinguish conspecific urine from conspecific “male water” and discriminated both between males and females and between conspecifics and heterospecifics. In contrast to female *X. birchmanni* from the Río Garces population, which prefer conspecific male chemical cues [16], female *X. birchmanni* from Huitznopala significantly preferred the urine of the congener *X. malinche* with which *X. birchmanni* naturally hybridizes [23]. Preference for heterospecific urine cues is consistent with heterospecific mating preferences observed throughout *Xiphophorus* for numerous traits [22], [27], [28]. Male Río Garces *X. birchmanni* prefer olfactory cues of female *X. malinche* over those of female conspecifics [19], and female *X. continens* prefer olfactory cues of male *X. montezumae* [21]. *X. pygmaeus* females, like the female *X. birchmanni* in this study, exhibit population-level differences in preferences for conspecific versus heterospecific traits [29].

Males target the release of their urine to maximize detection by receivers. Focal males substantially increase urination rate above baseline levels when other individuals are present, and specifically concentrate urination events to situations where others are spatially close. This ‘audience effect’ [3] indicates that the sexual cues are not released passively but emitted tactically with respect to intended receivers. We cannot rule out that males may increase urination near other stimuli besides conspecific females [30], but urine release does appear to be modulated by social cues. The nonrandom nature of urination may reflect costs of producing olfactory signals. Pheromone attractiveness is dependent on nutritional condition, suggesting that there may be physiological costs to pheromone production [25]. There may also be risks of having olfactory cues intercepted by potential predators [2]. Males, then, may enhance their signaling efficiency by structuring the temporal and spatial patterning of cue release so as to target attractive females in reproductive condition [19].

In addition to reserving urine for close interactions with females, males behave so as to optimize the transmission of signals in their physical environment. In both air [31] and water [32], fluid dynamics play a determining role in the transfer of chemical information from signaler to receiver. Swordtails live in shallow, rocky streams with moderate current flow [23], [33]. Signal transmission should be greatly enhanced if males are positioned upstream of females. Courting males spend the overwhelming majority of their time positioning themselves in such a way as to maximize pheromone delivery to females, maintaining their place directly upstream of females with respect to the prevailing current.

Animal signals are often structured to optimize the efficiency of transmission in their physical environment [34]. Our results show that male *X. birchmanni* control the spatial and temporal dynamics of sexual pheromone release in order to target intended receivers and to optimize transmission efficiency in the signaling environment. In order to minimize fluid loss, terrestrial animals must concentrate urine and release it in specific times and places [35]. This basic physiological constraint has facilitated the evolution of spatiotemporal control of chemical signaling in terrestrial taxa. Relatively free of this constraint, however, freshwater organisms urinate more or less continuously[36]. Thus, the remarkable degree of control over the release of chemical signals by male swordtails is likely the result of intense selective pressures on males to transfer important information directly to potential mates. Given the near ubiquitous nature of chemical signals in sexual communication, we suggest that tactical release of signals may be a widespread feature of chemical signaling in fishes. Further, the finding that chemosignals are released at specific times and places suggests that chemical signaling may constrain the expression of visual and mechanosensory signals used in courtship interactions. For example, optimal position in the current relative to the angle of sunlight and the visual background may be quite different from the optimal position relative to prevailing current. Given the potential for phenotypic and genetic manipulations of male signals in *Xiphophorus* [37], swordtails may constitute a powerful model for exploring multimodal interactions among sexually-selected traits.

## Materials and Methods

All swordtails were wild-caught adults from Hidalgo state, Mexico: *X. birchmanni* were obtained from the Río Atempa at Huitznopala [23] and *X. malinche* from the Río Claro at Tlatzintla, Mexico [38]. All animal work was approved by the Institutional Animal Care and Use Committee of Boston University (Protocol #04-040).

### (a) Urine visualization, collection, and preparation

To visualize and collect fish urine, we anaesthetized male *X. birchmanni* with the lowest concentration of tricaine methanesulfonate (MS-222) effective to cease all motor activity. Males were then injected intraperitoneally on each side of the body with approximately 100 µL of a fluorescein dye solution composed of 1 mg/ml fluorescein in physiologically buffered saline (PBS). Each injected male was placed in an 80 L tank adjacent to another tank containing five conspecific females of various sizes, adding 8 mL of Stress Coat (Aquarium Pharmaceuticals) to mitigate handling effects. Males typically resumed courting within five minutes. The male was allowed to court the females for sixty minutes under a 60 W ultraviolet (UV) light that caused the urine to fluoresce (see Video S1). Since swordtails are sensitive to UV light [39], males actively courted females in the adjacent tank even when primarily illuminated by UV light, allowing visualization of the release of the fluorescein dye in the context of normal sexual behavior. Fluorescein dye was clearly released through the vent, and no fluorescein was observed leaving the male through any other source. Every time urination was observed, a clean pipette was used to gently collect the fluorescing urine (and surrounding water) immediately upon release. This urine was placed into carbon-filtered, dechlorinated tap water at a concentration of one liter per male. To mitigate the effect of inter-male variation in signal quality, the urine of two males at a time was collected and combined for use as a stimulus in choice tests. As in previous studies of swordtail chemical cues, diluted urine was kept at room temperature and used in trials within 24 h of collection.

### (b) Female Preference Tests

We conducted standard dichotomous-choice mate-preference tests [16], [25] to test female *X. birchmanni* for species-specific responses to heterospecific versus conspecific male urine and for sex-specific responses to conspecific male versus female urine. After a 10-min acclimatization period, females were simultaneously presented with chemical cues on opposite sides of a 75 cm×20 cm aquarium filled to a depth of 20 cm. We recorded association time for 10 min, starting once a female had visited both sides of the aquarium; if the female failed to visit both sides within 5 minutes, the trial was aborted. Responses were analyzed using two-tailed Wilcoxon signed-ranks tests. To assay whether individuals attended to urine-borne stimuli, we conducted three-way choice tests in a Y-maze apparatus, consisting of three arms, 37 cm long×31 cm wide, extending from an equilateral triangle with 31-cm sides. Stimulus delivery and presentation protocol were identical to those employed for dichotomous tests, except that trial time was extended to 15 min and females were given 7.5 min to visit all three stimuli.

*X. birchmanni* females were presented with three chemical cues: 1) male urine collected during the fluorescein treatment, 2) “male water” that had housed conspecific males for 3 hours (containing urine and any other secretions), which has previously been shown to elicit species-specific responses [15], [16], [17], [18], and 3) carbon-filtered water as a negative control. For these trials, 5 mg of fluorescein was added to both the male water and the plain water to ensure the fish were not choosing or avoiding stimuli containing fluorescein. We conducted a Friedman repeated-measures nonparametric analysis of variance on female responses to the three stimuli, followed by *post hoc* WSR tests on pairwise responses to male urine versus “male water” and urine versus the blank control.

### (c) Social context and male urine release

To determine whether males urinate more often in close proximity to females, we monitored male urination patterns in the presence and absence of females. A fluorescein-injected male was placed in an 80-L aquarium marked lengthwise into four 12.5 cm wide quadrants. A 22-L “stimulus aquarium” abutted the observation tank. Each male was tested once in the presence of the visual cue of five female conspecifics in the stimulus aquarium, and once in the absence of any social cues. The order of presentation was alternated to control for order effects. In each trial, we observed the male for 60 minutes starting when the male resumed swimming. For every one-minute increment, we recorded the quadrant in which the male was located and whether or not a urine pulse was observed during that interval. If a male urinated twice in one minute, it was counted as two urinations. If a male was between quadrants, the quadrant where more than half of his body was located was the quadrant counted. We used WSR tests to evaluate whether (a) males spent more time in the quadrant closest to the adjacent tank (quadrant 1) when females were present; (b) whether males urinated more overall when females were present; and (c) whether males urinated proportionately more in the proximity of females, i.e. whether males urinated more per unit time spent in quadrant 1.

### (d) Spatial dynamics of courtship in the field

We used a Sony VX2000 digital video camera inside a Gates underwater housing to record courtship interactions in *X. birchmanni* – *X. malinche* hybrids at Calnali-mid [23], on the Río Calnali, Hidalgo. The camera was secured to the substrate, pointing directly into the prevailing current, and deployed for one hour. Naturally-occurring suspended particles in the water column allowed us to readily assess the direction of current flow in real time. We continuously monitored the videotape for male-female courtship interactions [40] and scored both total courtship time and the amount of time that males were positioned upstream of females, operationally defined as whether the male was oriented within approximately 60° of the prevailing current with respect to the female. We then conducted a one-sample *t* test versus the null expectation that one-sixth (60 out of 360°) of total courtship time should be spent upstream of the female.

## Supporting Information

Video S1 (WMV)
